# HEALTH CARE WORKERS’ PERSPECTIVES ON PROVIDING PALLIATIVE CARE TO MULTILINGUAL POPULATIONS WITH DEMENTIA

**DOI:** 10.1093/geroni/igae098.3573

**Published:** 2024-12-31

**Authors:** Katyayani Strohl, Neeta Ghosh, Syeda A Fatima Ahmed, Danae Dotolo, Rashmi Sharma

**Affiliations:** University of Houston, Houston, Texas, United States; University of Washington, Seattle, Washington, United States; University of Washington, Seattle, Washington, United States; University of Washington Medicine, Seattle, Washington, United States; University of Washington, Seattle, Washington, United States

## Abstract

**Importance:**

Persons living with dementia (PLWD) who prefer a language other than English (PLOE) are at high risk for sub-optimal palliative care. Although data suggest that multi-level (individual, clinical, healthcare system, community/policy-level) interventions may help address inequities, few studies have identified specific targets. Objective: To identify targets for multi-level interventions to improve palliative care for PLWD and PLOE.

**Methods:**

We conducted qualitative interviews with social workers and medical interpreters who work with Chinese, Spanish, or Vietnamese speaking PLWD. Interviews were conducted from October 2022-October 2023, audiotaped, transcribed, and coded by an interdisciplinary investigator team using an inductive iterative approach.

**Results:**

Interviews were conducted with 5 Spanish, 2 Chinese, and 2 Vietnamese interpreters and 5 social workers. Four themes emerged: 1) interpretation when the patient ‘wasn’t making sense’; 2) complexity with patient/family dynamics in decision-making; 3) barriers to anticipatory communication about dementia trajectory and quality of life; and 4) lack of culturally- and language appropriate resources. These data suggest multi-level intervention targets to improve palliative care in the context of dementia and linguistic diversity: 1) training interpreters about palliative care and how to navigate interpretation with PLWD, and providing clinicians communication skills training; 2) facilitating clinician-interpreter pre-meetings; 3) improving staffing and resources for social workers to shift from a crisis-focus to facilitating goals-of-care communication; and 4) increasing culturally and linguistically-appropriate and accessible resources. Impact: Our findings contribute to a gap in the literature about how to address the unique barriers PLWD with PLOE face, informing future intervention development.

